# Biophysical Cueing and Vascular Endothelial Cell Behavior 

**DOI:** 10.3390/ma3031620

**Published:** 2010-03-05

**Authors:** Joshua A. Wood, Sara J. Liliensiek, Paul Russell, Paul F. Nealey, Christopher J. Murphy

**Affiliations:** 1 Department of Surgical and Radiological Sciences, School of Veterinary Medicine, University of California, Davis, CA, USA; E-Mails: jawood@ucdavis.edu (J.A.W.); prussell@ucdavis.edu (P.R.); 2 Department of Chemical and Biological Engineering, School of Engineering, University of Wisconsin-Madison, Madison, WI, USA; E-Mails: sjlilien@students.wisc.edu (S.J.L.); nealey@engr.wisc.edu (P.F.N.); 3 Department of Opthalmology and Vision Sciences, School of Medicine, University of California, Davis, CA, USA

**Keywords:** endothelial, vascular, basement, membrane, pathomimetic, homeomimetic, nanotopography, biophysical, cues, modulus

## Abstract

Human vascular endothelial cells (VEC) line the vessels of the body and are critical for the maintenance of vessel integrity and trafficking of biochemical cues. They are fundamental structural elements and are central to the signaling environment. Alterations in the normal functioning of the VEC population are associated with a number of vascular disorders among which are some of the leading causes of death in both the United States and abroad. VECs attach to their underlying stromal elements through a specialization of the extracellular matrix, the basement membrane. The basement membrane provides signaling cues to the VEC through its chemical constituents, by serving as a reservoir for cytoactive factors and through its intrinsic biophysical properties. This specialized matrix is composed of a topographically rich 3D felt-like network of fibers and pores on the nano (1–100 nm) and submicron (100–1,000 nm) size scale. The basement membrane provides biophysical cues to the overlying VECs through its intrinsic topography as well as through its local compliance (relative stiffness). These biophysical cues modulate VEC adhesion, migration, proliferation, differentiation, and the cytoskeletal signaling network of the individual cells. This review focuses on the impact of biophysical cues on VEC behaviors and demonstrates the need for their consideration in future vascular studies and the design of improved prosthetics.

## 1. Introduction

Vascular diseases are the leading cause of death in developed countries [1,2]. Coronary heart disease alone is the cause of 1 in every 7 deaths worldwide [3,4]. In addition, more than 12 million people in the United States are treated for peripheral artery disease, and endothelial cell dysfunction is a known contributor to the progression of this disorder [2,5]. Pulmonary hypertension, another major vascular disease, is characterized by vessel wall distension which results in thickening of the wall and leads to decreased vessel compliance, narrowing, and occlusion [6]. There is a great need for an improved understanding of endothelial function. Elucidation of the interactions between vascular endothelial cells and the extracellular microenvironment will lead to improved strategies for the treatment of vascular disease.

Currently available treatments for vascular diseases depend on the severity of the diagnosis. In less severe cases, drug therapy in the form of vasodilators, diuretics, beta-blockers, statins [7], and a menu of other pharmaceutical therapies are used. In more severe disease states, surgical grafts are often necessary [3,4,8,9]. In cases where vessel grafting is required, autologous tissues are preferred. However, 7% of patients do not have suitable tissue for harvest due to widely distributed disease progression [10,11,12,13]. In cases where autologous transplants are not available, several alternative options including heterogenic donation or synthetic vascular implants are employed [14]. However, heterologous grafts have limitations with potential immunologic response in the patient and insufficient donor availability. There is a demonstrable need for synthetic vascular replacements with optimal biointegration and performance parameters.

Currently, there are a variety of synthetic vascular implants available for high flow large vessels developed from Dacron and other synthetic polymers. The failure rate for small vessels (less than 5 mm in size) remains high due to thrombosis and resulting occlusion of the graft [15,16,17]. The direct involvement of vascular endothelial cells (VECs) in vascular disease has been demonstrated and the establishment of a functional endothelium in prosthetics is critical to their success [3,7]. Graft failure is primarily due to a lack of endothelium and differences in stretch, local compliance, and vascular resistance (the change in diameter of a vessel under pressure) between the native vessel and the graft. Graft failure can lead to progression of atherosclerotic disease, thrombosis, or hyperplasia [14,18,19]. To improve clinical success rates of vascular grafts, strategies that make the graft more “biomimetic” in terms of surface chemistry and mechanical attributes are being explored. One approach is to mimic the native biophysical features which directly impact endothelial cell function and homeostasis.

The importance of the mechanical properties of vessels and their direct impact on disease has been known for decades [20]. Alterations in the biophysical microenvironment of endothelial cells, which include the underlying basement membrane, have been implicated in the homeostatic state of the vascular tissue leading to various vascular pathologies [21,22,23,24,25]. Vascular scaffolds for guided tissue regeneration have been developed but the biophysical features including topography and compliance of the native vessel have not yet been fully incorporated into prosthetic design and their absence may be a contributing factor to prosthetic vessel failure [14,26]. Topographic features of the vascular basement membrane from several different anatomic sites have been characterized and been found to range in size from the nano to submicron scale [27]. In addition, the thickness of the basement membrane of different vascular tissues can vary significantly. This observation suggests that the variations in thickness have an impact on another biophysical feature, compliance [28]. The effect of compliance on endothelial behavior has been observed in vascular angiogenesis as well as in endothelial cell migration, adhesion, proliferation, and differentiation [29,30,31,32,33,34]. These biophysical cues affect the endothelial cells at the molecular level. Ongoing work from our laboratory have shown that 4,000 genes in Human Umbilical Vein Endothelial Cells (HUVEC) were found to exhibit a greater than 2 fold change in expression (up or downregulated) when cultured on sub-micron to nano-topographically patterned surfaces as compared to flat surfaces [35]. The combined effects of these extracellular cues suggest that incorporating these attributes into prosthetic design may favorably impact endothelial cell function and graft performance. The focus of this review is to demonstrate the need for the consideration biophysical cues, such as topography and compliance, in future vascular studies and the design of improved prosthetics based on the evidence that these cues have a critical impact on VEC behaviors.

## 2. Vascular Endothelial Cells

The proper functioning of the vascular network within the human body is essential for every cell, tissue and organ. Blood vessels are involved in the distribution of oxygen and diffusion constraints limit the distance of nearly every cell to be within 100–200 µm from a blood vessel [36]. In capillary beds the circumference of the vessel can contain as little as one endothelial cell [37]. Intrinsic to vessel structure are vascular endothelial cells and their associated basement membrane as well as the stromal elements of the vessel wall including smooth muscle cells [36,38]. This simple squamous epithelium was historically thought to be little more than a barrier; studies have since demonstrated the complex functionality and heterogeneity of these cells [39]. The rich diversity of endothelial cells is due to a complex network of endothelial progenitor cells that give rise to the various endothelial cell types including a circulating population [40,41]. VECs line the vessels of the body and are responsible for the correct trafficking of chemical signals and immune cells [42]. Additionally, VECs provide structural support to the vessel [24,43]. Chemical “communication” between VECs is done through intercellular junctions including caveolae [39]. Vasculogenesis, the process of new blood vessel formation, is the result of migrating and subsequently proliferating endothelial cells induced by physical and chemotactic signals [44,45,46,47,48,49,50,51]. The formation of new blood vessels is critical for development, and normal adult processes such as wound healing. New blood vessel formation also characterizes many pathologic states including cancer metastasis [52,53,54]. Recent studies have shown that the contractile forces generated by the VEC cytoskeleton in conjunction with matrix stiffness and biochemical cues regulate vasculogenesis [55].

In addition to regulating vasculogenesis, biophysical cues also regulate resistance to physical stresses. VECs undergo a great deal of mechanical stress when subjected to shear flow or pulsate stress [56,57,58,59,60]. To resist these stresses, biophysical and biochemical signals initiate remodeling of the VEC cytoskeleton to form actin stress fibers [6,56,57,58,61]. VEC cytoskeletal structure and organization largely determines their intrinsic biophysical properties. The endothelial cell cortex contains a cytoskeletal meshwork of two different size scales. The larger meshwork is composed principally of actin fibers forming pores ranging from 0.5–1.0 µm^2^. Intertwined within the larger network is a smaller meshwork (<0.5 µm^2^) that is less rigid than the larger meshwork [62]. The combined rigidity of these two meshworks are thought to be responsible for the local compliance (Young’s modulus) of the individual endothelial cells. Thus, the two meshworks are thought to be responsible for the Young’s modulus of the cell; that is, the resistance to deformation or the rigidity of the cells in response to external forces such as blood pressure. The unit of measure of Young’s modulus is the Pascal. The local compliance of endothelial cells also varies depending on the spatial location along the cell from which the measurement is obtained. Values for Young’s modulus (compliance) from different areas of the endothelial cell have been obtained and are reported to be approximately 5–7 kiloPascals (kPa) in the region of the nucleus and 0.32–3 kPa in the cytosolic region [42,43,59,63,64,65]. Endothelial cells are exposed to shear stress from fluid flow *in vivo* and measurements of the local compliance of endothelial cells under these conditions demonstrate a 2-fold decrease in compliance (becomes stiffer) [56]. The aforementioned results support the idea that the mechanical properties of endothelial cells play an important role in the vascular permeability between the tight junctions of the cells and resistance to shear stress from fluid flow [43,61]. These endothelial cell properties are essential for the strength and integrity of the vessel and their regulation by biophysical cues demonstrate the importance of extracellular biophysical cues in both native vessels and prosthetic design.

**Figure 1:**
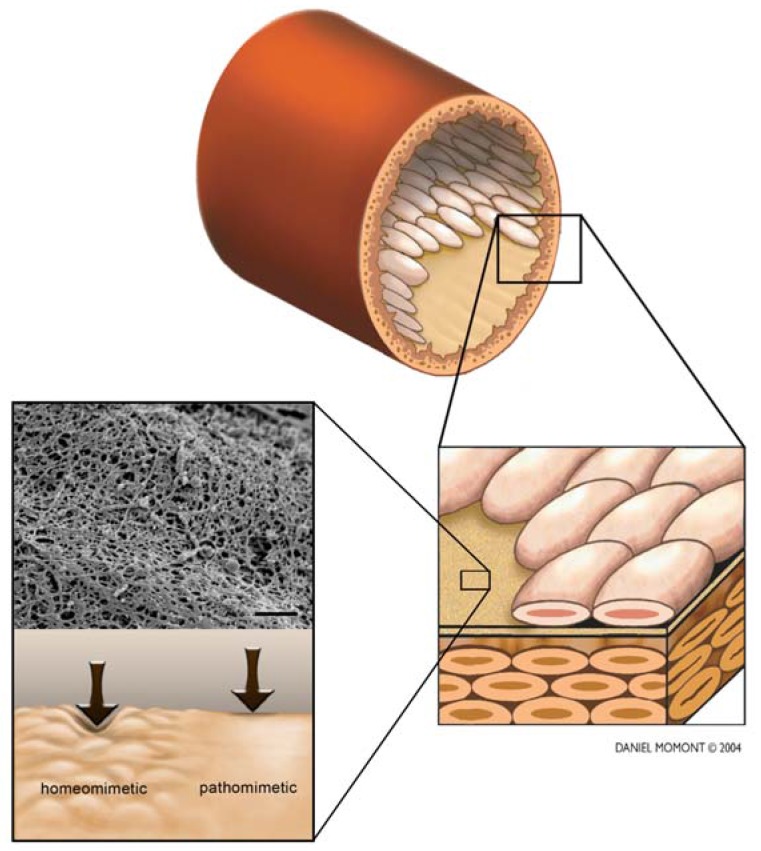
Basement membrane topography and compliance.

Biophysical cues also play an important role in disease states, VEC failure in the form of aberrant permeability, rigidity, or receptor expression is related to several diseases including ischemic heart disease and atherosclerosis [5,66]. Collapse of the endothelial cell cytoskeleton can lead to vessel collapse [24]. In response to hypertension, VECs are capable of remodeling the vascular wall [25]. Onset of atherosclerosis is a consequence of interactions among the smooth muscle cells, endothelial cells, and monocytes (which have been shown to decrease the compliance and adhesiveness of endothelial cells) contributing to an increase in deformability [67,68]. Furthermore, response by the endothelium following injury (or disease states) can lead to increased secretion of basement membrane proteins and basal lamina hyperplasia [66]. In aberrant conditions, inappropriate cues due to deformation of the basement membrane are associated with several vascular disorders including muscular dystrophy, skin disorders, and kidney defects [21,22,69]. In aggregate, these data lead us to the hypothesis that the local compliance of the stromal elements of the vessel (possibly including the basement membrane) is decreased in certain disease states and that this change in local compliance directly participates in disease progression (Figure 1). 

The basement membrane is a rich felt-like meshwork containing pores and fibers arranged in an isotropic manner with feature sizes ranging from the submicron level (100–1000 nm) to the nanoscale (1–100 nm). The impact of simulating biomimetically relevant topographic features has been shown to change over 4,000 genes more than 2-fold up or down compared to flat surfaces. Substratum topography has been shown to modulate cell adhesion, migration, proliferation, and differentiation. Additionally, the presence of topographic cues can modulate the response of cells to cytoactive molecules present in the soluble signaling environment. The local compliance of the basement membrane has been reported to be between 8–35 kPa. Changes in compliance have been demonstrated to impact cell morphology, differentiation, migration, and compliance. In disease states such as atherosclerosis the basement membrane has been shown have increased thickness in some vessels. Additionally, endothelial cells have been shown to secrete basement membrane components and the overall vessel is less compliant (and thus stiffer and more rigid and resistant to external forces such as blood pressure) in some disease states. The combination of this evidence leads us to the hypothesis that the compliance of the stromal elements of the vessel (possibly including the basement membrane) is decreasing in certain disease states and this change in compliance directly participates in disease progression. Furthermore, the use of pathomimetic substrates which appropriately simulate the increased rigidity observed in some disease states will lead to a better understanding of the molecular mechanisms behind basement membrane remodeling in changes in endothelial cell morphology (Scale bar = 600 nm).

The biophysical properties of endothelial cells can be altered by disease as well as by therapeutic agents. One example is evident in atherosclerosis where secretion of oxidized low-density lipoproteins lead to increased VEC stiffness and dysfunction [65]. In addition, the secreted lipoproteins deposit within the underlying basement membrane which we hypothesize alters the biophysical properties of the matrix initiating further changes in the overlying endothelial cells in a feedback loop mechanism [39]. This evidence suggests that a complex feedback loop between the endothelial cells and the biophysical attributes of the basement membrane may be occurring resulting in either maintenance of homeostasis or disease progression. New developments in the treatment of heart disease utilize mineralocorticoids or glucocorticoids which have been shown to cause enlarged, stiffened cells or strengthened cell-cell contacts, respectively [64]. Therapeutic interventions with hormones such as 17β-estradiol has been shown to increase endothelial cell volume, surface area, and elasticity through inhibition of Na^+^/H^+^ exchange thus providing protective effects against the onset of atherosclerosis [70]. Both the presence of disease and many therapeutic interventions impact the biophysical properties of endothelial cells. A better understanding of the regulation of these properties would lead to novel insights into both disease progression and improved treatments.

In contrast to the aberrant response of endothelial cell in disease states, providing biomimetic biophysical cues will likely promote normal endothelial cell behavior and the migration of endothelial progenitor cells into a transplanted vessel [71]. Once the graft is populated, endothelial progenitor cells can provide a native endothelial cell lining in addition to secreting basement membrane and signaling molecules for the subsequent trafficking of cytoactive factors [7,71,72,73]. Overall, endothelial cells “sense” both the topography and local compliance of their extracellular microenvironment, represented primarily by the basement membrane and actively respond to changes therein [29,30,74].

## 3. Basmenet Membrane

The basement membrane is a specialization of the ECM secreted by the endothelial cells themselves [75,76]. Found basolaterally to all endothelial cell monolayers [77], the native basement membrane of VECs is a complex, heterogenic mixture of collagen IV and V, laminin, enactin/nidogen, fibronectin, the heparin-sulfate proteoglycans perlacan, and an assortment of other proteins present at lower levels [22,28,74,76,78,79,80,81,82,83,84]. The vascular basement membrane is found on the periphery of the tunica intima next to the tunica media which contains the smooth muscle cells [85]. The combination of the chemical and physical cues, provided by the self assembled layer of proteins that compose the basement membrane and supporting cells (such as smooth muscle cells), are major elements of the VEC microenvironment [86,87,88].

The composition of all basement membranes includes the basal lamina, which includes the lamina lucida (15–65 nm thick) and lamina densa (15–125 nm thick). There is one report suggesting that the lamina lucida may be a dehydration artifact from fixation but a consensus has not been reached thus far [89]. In some tissues a lamina fibrorecticularis (2–15 µm) is also described [78,82,90,91,92] The basement membrane self assembles to provide support for the overlying endothelial cells as well as providing the VECs a variety of biochemical cues [22,28,76,79,82]. The VECs are located over a thin basal lamina which in small vessels or capillaries encloses the pericytes [38,84]. In larger vessels this thin layer separates the VECs from the smooth muscle cells [84]. VECs anchor to, and interact with, the basement membrane and ECM through focal adhesions which are composed of integrin, talin, vinculin, α-actinin, and other proteins [28,76]. While several mechanisms may be involved, the formation and maintenance of the focal adhesions is regulated by Rho and the corresponding signaling cascade [76,93]. Basement membranes also serve as a reservoir of cytoactive factors including growth hormones and other signaling molecules [76,94].

In addition to soluble and surface associated biochemical cues, the basement membrane provides a variety of biophysical cues [28,78,82,95]. Topographically, the endothelial basement membrane is comprised of nano and submicron scale features arranged in a felt-like meshwork [28,96]. The local compliance (Young’s modulus) of the native basement membrane of vasculature is between 8–70 kPa, though some researchers have reported values greater than 100 kPa and one report is in the MPa range, though this tissue was frozen prior to analysis and the reported modulus is not consistent with the other literature [23,97,98,99,100]. These discrepancies may be the result of vascular stiffening or the differences in the acquisition and analysis of atomic force microscopic data. Reported values for the compliance of the human corneal basement membranes range from 2–80 kPa suggesting that the compliance for basement membranes is generally 100 kPa or lower [75].

Biophysical cues also participate in the initiation and cessation of angiogenesis. During angiogenesis the basement membrane is degraded by matrix metalloproteinases limiting the presence of collagen type IV [101,102]. The ECM, containing a high laminin content [101], stretches out and its increased compliance promotes migration and proliferation [30]. Additionally, this scaffold provides critical biophysical and organizational cues to endothelial cells during migration into vessel tips [95,101].

## 4. Biomaterials

Several attempts have been made to replicate the biophysical features of basement membranes. Matrigel is a commercially available product that is commonly used as a basement membrane replacement. While it has yet to be fully defined, it is derived from a mouse tumor model and is considered basement membrane-like [77,103]. Our laboratories have documented Matrigel to posses topographic features and intrinsic compliance very similar to those found for native basement membranes. The reported modulus of Matrigel ranges from 120–450 Pa depending on whether the value was measured at or below 37 °C with the softer values in overall compliance measured at lower temperatures where Matrigel begins to loose its gel characteristics [103]. The topographic features of Matrigel have been defined and quantitated using SEM and are similar to those of native basement membranes [27,103]. Matrigel has also been combined with silk based electrospun fibers to simulate biomimetic mechanical properties and topography [104].

Decellularized, sterilized, explants have also been used as vascular scaffolds in mice. No immune response was detected and the grafts appear to remain patent, however, the explants were not tested long term [1]. Other efforts to incorporate topographic cues from biological sources have employed donor derived amniotic basement membranes from heterogenic sources [105]. Substantive research has also been performed on collagen gels to serve as mimics of ECM [106]. Synthetic surfaces such as polyvinyl alcohol gels have also been engineered with organic protein coated surfaces to serve as prosthetic membranes and these mimic some of the elastic characteristics of vessels [107]. To date, an optimal biomaterial for use as a vascular graft has not been identified. The cause of failure of previous efforts is multifaceted including the lack of integrating biomimetic topographic and compliance cues.

## 5. Topography 

Basement membranes possess a rich topography consisting of holes, fibers, and bumps arranged in a felt-like structure [27,28,76,77,96,108,109] The effects of micron scale topographic cues on endothelial cell proliferation, adhesion, and migration under shear stress and non-stress conditions have been previously reported [110,111,112,113]. Individual topographic features of native basement membranes, however, are much smaller, residing in the nano-submicron range. While micron scale topography does impact cell behavior, these size scales are too large to be biomimetically relevant and submicron to nanometer scale features have demonstrated a greater impact on cell behavior. The effects of nanotopographic cues have been shown to be critically involved in cell migration, proliferation, adhesion, and protein and gene expression [78,114,115,116,117,118,119]. The influence of topography has even been shown to regulate differentiation [120,121,122]. Efforts to study nanotopographic cues in order to replicate native basement membrane and eventually improve vascular prosthetics have only recently been reported [3,78,105,123,124,125,126,127,128]. In addition, most prosthetics have included topographic features which are much larger (greater than 1 um) than that found in the native VEC basement membrane.

As stated earlier, endothelial cells have been shown to react to biophysical cues with changes in cell behavior. Human endothelial progenitor cells studied on anisotropically ordered ridge and groove patterned substrates were found to exhibit changes in orientation, migration, alignment, and reduced proliferation [129]. Similar studies performed by our laboratory and others on cell types other than VEC have found that cell culture on nanotopographically patterned silicon wafers impacts cell adhesion [128], migration [130], proliferation [131], orientation, alignment [132], and differentiation [116,122,127]. In aggregate, these studies have demonstrated that topographically patterned surfaces containing features in the nano-submicron range profoundly influence a broad menu of cell behaviors and these features are critical in the design of improved vascular prosthetics.

Metal-based and other synthetic surfaces including nickel-titanium and titanium oxide alloys have been used in a number of studies to produce vascular stents with the incorporation of topographic features [3,124,125,133,134,135]. Furthermore, studies using nickel-titanium surfaces with isotropically ordered submicron to nanometer features have demonstrated that endothelial cells align in a similar pattern to natural endothelium [3]. Studies using calcium phosphate ceramic particles found that varying the micron level size of the particles and the particle shape controlled the differentiation of endothelial progenitor cells [136]. Polyethylene glycol chains have also been employed to create nanometer scale roughness on smooth surfaces and it was found that surface features on the scale of 10–100 nm improved both cell adhesion and proliferation [126]. Electrospinning of polyurethane and other polymers have successfully simulated ECM topography’s submicron scale but have not successfully incorporated biomimetic values of local compliance to date [137]. A number of these studies have concluded that sub-micron to nanoscale topographic features promote endothelial cell adhesion, migration, and proliferation [3,124,125,129,133,134].

Our group has employed reactive ion etching and electron beam lithography to simulate the various topographic features of the basement membrane and tested a variety of cell types on these surfaces including endothelial cells [27,75,76,78,131,138,139]. Additionally, we have shown that the impacts of topographic cues are augmented by the presence of the soluble chemical cues found in serum [139]. Cells on anisotropically ordered ridge and groove patterned surfaces exhibited contact guidance, and cellular alignment which was not observed on isotropically ordered surfaces with holes [76,115,128,139]. Cell shape has been shown to be controlled by topographically patterned surfaces and control of cell shape has been shown to regulate cell fate [76,114]. Surface feature depth was also shown to induce greater alignment response on feature depths ≥300 nm [76,78,114]. The shape of the feature, independent of feature size, has also been shown to impact cell alignment, migration, and focal adhesion formation and size [27,115,128,131,138,139].

## 6. Compliance

The *local compliance* of the basement membrane is quantified by Young’s modulus, which describes the resistance to deformation of the substrate that the endothelial cells are in contact with [75,97,98,99,140,141]. Local compliance is distinguished from the term vascular resistance which refers to the change in diameter of a vessel under pressure [18,25]. Burst pressure resistance has also been engineered into current vascular prosthetics, and is referred to as compliance by some authors [142]. While resistance to both types of stress is important, few prosthetics include local compliance as a critical design parameter. The local compliance of the basement membrane is an important biophysical cue that modulates the migration, proliferation, differentiation, adhesion, and membrane organization of endothelial cells [32,33,74,75,119,143,144,145,146,147]. The majority of *in vitro* compliance studies have employed polydimethylsiloxane (PDMS), polyacrylamide, alginate, or agarose [143]. Recent reports have demonstrated that aortic smooth muscle cells spread and organize their cytoskeleton to a greater degree on stiff gels (modulus of 66 kPa or greater) compared to softer gels (modulus of 22 kPa or less [32,146]. Endothelial cells have been shown to change their cellular functions in response to matrix stiffness [148,149]. Increasing matrix stiffness has also been shown to decrease vascular network formation [148].

The behavior of endothelial and endothelial progenitor cells on compliant surfaces has yet to be fully explored. However, in the use of mesenchymal stem cells (MSCs) to repair infracted heart tissue it was found that myosin/actin striations form on gels with a normal muscle Young’s modulus (~12 kPa) but not on softer or stiffer gels simulating dystrophic muscle [33]. Further studies using MSCs found that even softer gel environments (elastic modulus ~1 kPa) lead to differentiation of MSCs into neurogenic lineages, moderately stiff gels lead to myogenic lineages (elastic modulus ~10 kPa), and stiff gels (elastic modulus ~ 100 kPa) lead to osteogenic lineages [147,150]. Studies combining MSCs and endothelial cells found that mechanically straining the MSCs increased endothelial cell proliferation [151]. Other studies have found that combining MSCs with endothelial cells in stiff fibrin matrices results in vessel formation with increased compressive stiffness [152]. Studies have also employed nano-particles to decrease the compliance of simulated basement membrane [153]. The accumulated evidence suggest that the biophysical cues provided by different compliant environments have a powerful impact on the differentiation potential of endothelial progenitor cells contribute to the heterogeneous nature of the vascular endothelial cell populations in different vessels sizes and types.

## 7. Conclusions

Local basement membrane compliance and topography are important intrinsic biophysical attributes of the microenvironment of vascular endothelial cells (VECs). These properties participate in the maintenance of homeostasis and likely contribute to the pathogenesis of disease states. A growing body of evidence documents VEC behaviors to be profoundly modulated by these biophysical cues. The use of synthetic matrices with biophysical attributes that mimic the normal native (homeomimetic) and disease (pathomimetic) states of vessels will contribute to our understanding of VEC biology in health and disease. The integration of topographic cues and local compliance has been shown to dramatically influence nearly every aspect of endothelial cell behavior. The combined findings that topography and local compliance play such a central role in VEC behavior (*i.e.,* differentiation, adhesion, proliferation, migration, and cytoskeletal organization) suggests that incorporating both of these biophysical attributes into vascular prosthetic design and fabrication will lead to improved performance.
